# A Rare Case of a Good Neurological Outcome following Traumatic Foix-Chavany-Marie Syndrome

**DOI:** 10.1155/2024/6652867

**Published:** 2024-05-10

**Authors:** Katalin Arki, Christian Degen, Philipp Gruber, Luca Cioccari

**Affiliations:** ^1^Department of Intensive Care Medicine, Kantonsspital Aarau, Aarau, Switzerland; ^2^Department of Diagnostic and Interventional Neuroradiology, Kantonsspital Aarau, Aarau, Switzerland

## Abstract

Traumatic brain injury (TBI) can have profound acute and chronic effects, leading to permanent disabilities and diminished quality of life. Pseudobulbar palsy and its infrequent subtype, Foix-Chavany-Marie Syndrome (FCMS), represent rare complications of TBI, manifesting as deficits in craniofacial motor function and automatic-voluntary dissociation. We present a case of a 58-year-old male who developed FCMS following severe TBI from a cycling accident. Initial imaging revealed extensive brain injury with subsequent development of FCMS characterised by bilateral cranial nerve dysfunction, notably facio-pharyngo-glosso-masticatory diplegia with preserved automatic motor function. This case contributes to the limited literature on traumatic FCMS, highlighting its distinct clinical features and potential for favourable outcomes compared to nontraumatic cases. Early recognition and comprehensive management, including supportive therapy and addressing underlying conditions, are paramount for optimising patient outcomes.

## 1. Introduction

Traumatic brain injury can result in a wide range of acute as well as chronic consequences that can lead to permanent disabilities, high mortality rates, and reduced life expectancy [1, 2]. TBI can also lead to a variety of secondary pathological conditions affecting different parts of the brain. Such conditions include focal neurological deficits, cognitive impairment, seizures, sleep disorders, neurodegenerative diseases, and dysregulation of neuroendocrine function [3].

Pseudobulbar palsy is usually caused by bilateral damage to the corticobulbar pathways, which are upper motor neuron pathways that run from the cerebral cortex to the cranial nerve nuclei in the brainstem. Patients have trouble chewing and swallowing and have increased reflexes and spasticity in the tongue and bulbar region, slurred speech (which is often the initial presentation of the disorder), and sometimes uncontrolled emotional outbursts. Possible causes of pseudobulbar palsy include neoplasms, inflammatory diseases, demyelinating diseases, multiple and recurrent strokes, and traumatic brain injuries [4].

Foix-Chavany-Marie Syndrome (FCMS), also known as anterior opercular syndrome, is a rare type of pseudobulbar palsy causing facio-pharyngo-glosso-masticatory diplegia with automatic-voluntary dissociation [5]. Automatic-voluntary dissociation is a paresis of voluntary movements as opposed to automatic movements and is a defining characteristic of FCMS, highlighting the divergent corticobulbar pathways of voluntary and automatic control of the facial, pharyngeal, lingual, and masticatory muscles [6, 7]. Patients are unable to open or close their mouth voluntarily but are able to perform automatic actions such yawning or smiling. FCMS is most associated with cerebrovascular disease; however, it may also be caused by central nervous system infections, developmental disorders, neurodegenerative disorders, and, rarely, trauma [8–12].

FCMS usually leads to significant long-term neurological impairment. In this report, we present a rare case of a patient suffering from FCMS after traumatic brain injury with a good neurological outcome.

## 2. Case Presentation

**Figure 1:**
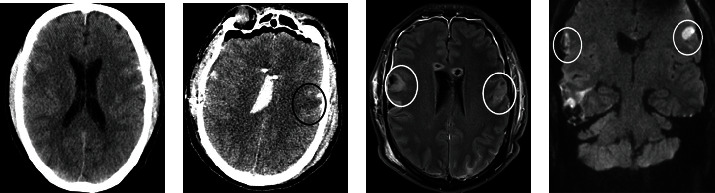
(a) Initial CT scan showing acute traumatic changes. (b) The postoperative CT revealed a subacute ischaemic lesion on the left operculum (black circle) and residual post-traumatic and postoperative changes. On the T2-weighted MR images (3T Magnetom Vida, Siemens, Erlangen, Germany), (c) hyperintense lesions in both opercula (white circles) are detected which are due to parenchymal defect caused by contusion on the right side and due to an ischaemic event on the left side, as clearly seen in the (d) diffusion-weighted images (DWI).

A right-handed 58-year-old male patient sustained a severe traumatic brain injury following a cycling accident and was admitted to our level one trauma centre. His initial prehospital Glasgow Coma Scale (GCS) was 15. Upon admission, the patient's GCS rapidly decreased to 12; he was unable to speak, developed anisocoria, and became increasingly disoriented and agitated. He was subsequently intubated in order to aid computed tomography (CT) imaging, which showed Marshall [13] grade II traumatic brain injury with a right-sided subdural haematoma and bilateral subarachnoid bleeding as well as a secondary right-sided parietal parenchymal haemorrhage (Figure 1(a)) with a subsequent midline shift to the left and tonsillar herniation. Extracranial injuries included multiple rib fractures and a pubic branch fracture with minor active bleeding that were managed conservatively.

The patient underwent immediate craniotomy, haematoma evacuation, and bilateral external ventricular drainage (EVD) placement. Following postoperative extubation, the patient was well but was still unable to verbally communicate. On further neurological examination, we observed bilateral dysfunction of cranial nerves V, VII, IX, X, and XII, with isolated facio-pharyngo-glosso-masticator diplegia. The patient was not able to close his mouth, chew, or initiate swallowing, and the gag reflex was diminished. He could, however, comprehend spoken and written language. The tongue was immobile but did not deviate. No signs of vocal cord paralysis were observed. Automatic motor function of the aforementioned cranial nerves remained intact, and the patient was observed spontaneously smiling and yawning.

A follow-up CT scan was performed postoperatively showing a diffuse cerebral oedema with deep standing cerebellar tonsils, but with a regressive midline shift, and a subacute ischaemic lesion on the left operculum and post-traumatic changes (Figure 1(b)).

Magnetic resonance imaging showed focal T2w hyperintensities in both opercula due to parenchymal damage caused from traumatic contusion on the right side and from an ischaemic event of undetermined cause on the left side with hyperintense signals in the diffusion-weighted imaging (DWI) with corresponding hypointense values in the apparent diffusion coefficient (ADC) (Figures 1(c) and 1(d)). Based on the neurological deficits, in combination with the supporting radiological findings of bilateral isolated lesions in the region of the anterior operculum, a diagnosis of FCMS was made.

The central management revolved around intensive speech and language therapy, continued nasogastric feeding until the ability to swallow was sufficiently restored, and careful positioning of the patient due to the high risk of aspiration. The patient improved gradually, and by day 28 postinjury, he was transferred to a rehabilitation centre, with only mild residual short-term memory disturbance, mild dysarthria, and fully recovered craniofacial motor function. At 12 months, the patient's Extended Glasgow Outcome Scale (GOSE) was 6/8, indicating a favourable outcome [14]. There was still a mild to moderate cognitive impairment as well as learning and memory disabilities. The patient was able to manage activities of daily life at home, but reintegration into the workplace at full capacity was not yet possible due to the residual neurological deficits.

## 3. Discussion

There are three main subtypes of pseudobulbar palsy, based on the anatomical location of the lesion: cortical, basal ganglia/capsular, and brainstem/cerebellar. FCMS is a cortical subtype of pseudobulbar palsy. The syndrome is named after the neurologists Charles Foix and Jean Alfred Émile Chavany and paediatrician Julien Marie who described it in 1926 [15]; however, the syndrome was first characterised by Magnus in 1837 [16].

It can be distinguished from other subtypes of pseudobulbar palsy by the absence of urinary dysfunction, emotional incontinence, and muscle tone abnormalities [17]. Moreover, FCMS does not produce fasciculations or atrophy of the tongue, and unlike neuromuscular junction disorders such as botulism and myasthenia gravis, patients maintain involuntary innervation, normal eye movements, and intact brainstem reflexes [18, 19]. In children presenting with a congenital form of FCMS, there is a clinical overlap and common pathogenesis with other syndromes such as Worster-Drought syndrome [20]. The differential diagnosis of cranial nerve pathologies is diverse, and a thorough knowledge of their anatomy and function is fundamental to facilitate the diagnosis of this disorder [21, 22].

In a review of 62 cases of FCMS, Weller described 5 distinct clinical types based on pathogenesis [5]. The most frequent aetiology was thrombotic or embolic cerebrovascular disease, followed by central nervous system infections. Other causes included a neurodevelopmental disorder, a reversible form that occurs in children with epilepsy, and finally a neurodegenerative form. Management of these patients is dependent on aetiology and requires a multidisciplinary approach. Irrespective of aetiology, early speech therapy and oral motor exercises are essential. As swallowing is affected, management by means of nasogastric or percutaneous endoscopic gastrostomy feeding should be considered and minimising the risk of aspiration is crucial [23, 24]. An MRI should be performed if there is a strong clinical suspicion, as findings on CT are sometimes subtle or difficult to find [25].

We found only 5 other cases of traumatic FCMS published to date (Table 1). Two of them incurred unilateral lesions [8, 12], two had bilateral lesions [10, 11], and one had sequential head injuries [9]. The neurological outcome in these cases of post-traumatic FCMS was good or mildly impaired in 3 patients [8–10], while 2 patients had poor neurological recovery [11, 12]. Of note, poor recovery is consistently reported in nontraumatic cases [5]. It appears that FCMS patients with traumatic brain injury have a more favourable prognosis than those with other aetiologies and often recover at least partially. This may be because traumatic brain swelling is likely to be more reversible than ischaemic lesions. However, due to the rarity of the syndrome, there are no generalisable figures on the outcome.

In conclusion, acute onset facio-pharyngo-glosso-masticatory diplegia with automatic-voluntary dissociation is a characteristic feature of FCMS—a rare type of pseudobulbar palsy. The condition has multiple causes and variable long-term outcomes. However, traumatic FCMS seems to have a better prognosis than nontraumatic aetiologies. In cases where there is a strong clinical suspicion, an MRI should be performed as CT findings may be subtle or lacking. Early recognition and management of the underlying condition appear to be key factors. Supportive therapy including speech therapy, facilitation of adequate nutrition, and reducing the risk of aspiration is essential.

## Figures and Tables

**Table 1:** Summary of prior reported cases of traumatic Foix-Chavany-Marie Syndrome.

	Reference	Age	Sex	Mechanism of trauma	Lesion	Symptoms	Treatment	Recovery
1.	Laurent-Vannier et al. [11]	10	Male	Car accident	Bilateral	Automatic-voluntary dissociation. Anarthria. Loss of voluntary control of muscles supplied by nerves V, VI, IX, X, and XII.	Surgical removal of extradural haematoma	Poor
2.	Campbell et al. [9]	24	Male	Assault	Sequential	Dysarthria. Bilateral, upper motor neuron facial weakness, swallowing difficulties, loss of gag reflex, and no voluntary movements of tongue.	1. Left side: surgical evacuation 2. Right side: conservative	Good
3.	Nitta et al. [12]	20	Female	Car accident	Unilateral	Automatic-voluntary dissociation. Loss of speech and bilateral loss of voluntary control of muscles supplied by cranial nerves V, VII, IX, X, and XII.	Surgical removal of haematomas	Partial
4.	Acioly et al. [8]	28	Female	Penetrating injury	Unilateral	Automatic-voluntary dissociation. Loss of voluntary control of muscles innervated by cranial nerves V, VI, VII, IX, X, and XII.	Decompressive craniectomy	Full
5.	Digby et al. [10]	62	Male	Cycling accident	Bilateral	Dysphasia. Bilateral lower face, tongue and pharyngeal paralysis, bilaterally abducted vocal cords, and limited movement of forehead.	Conservative with ICP monitoring	Good

## Data Availability

Data supporting the conclusions are available from the corresponding author upon reasonable request.
